# Surface Modification of Aged Steel Slag Aggregate and the Road Performance of Asphalt Mixtures

**DOI:** 10.3390/ma19102031

**Published:** 2026-05-13

**Authors:** Yaoting Zhu, Qi Xiong, Yuqi Liao, Xin Yu, Chunpeng Yan

**Affiliations:** 1 Jiangxi Communications Investment Maintenance Technology Group Co., Ltd., Nanchang 330200, China; zhuyt1982@163.com; 2 Jiangxi Key Laboratory of Highway Bridges and Tunnels, Nanchang 330200, China; 3 School of Civil Engineering, Chongqing Jiaotong University, Chongqing 400074, China; 13367916409@163.com; 4 Jingdezhen Road and Bridge Construction Group Co., Ltd., Jingdezhen 333003, China; 13970374552@163.com; 5 College of Civil and Transportation Engineering, Hohai University, Nanjing 210098, China; yuxin2009@hhu.edu.cn

**Keywords:** aged steel slag, surface modification, aggregate properties, asphalt mixtures, road performance

## Abstract

**Highlights:**

- Surface modification of aged steel slag (ASS) using epoxy asphalt-based organic rejuvenator (EAOR) effectively optimizes its aggregate characteristics.
- Asphalt mixtures incorporating EAOR-modified ASS demonstrate significantly enhanced moisture damage resistance and pronounced elastic benefits.
- This technical approach enables reduced asphalt binder content during mixture compaction while increasing the volumetric replacement rate of steel slag, thereby contributing to the mitigation of natural aggregate scarcity.

**Abstract:**

Surface modification processes significantly influence the aggregate characteristics of steel slag and the road performance of asphalt mixtures. Therefore, this study employs four different substances to conduct surface modification treatment on aged steel slag with particle size ranges of 4.75–9.5 mm and 9.5–13.2 mm to determine the optimal surface modification process through macroscopic and microscopic analytical methods. Ultimately, a comparative analysis is performed on the pavement performance of asphalt mixtures incorporating aged steel slag and modified aged steel slag at different volume replacement ratios. Experimental results demonstrate that Epoxy Acrylate-Modified Organosilicon Resin (EAOR) significantly improved the aggregate properties of Aged Steel Slag (ASS), making it a viable alternative to natural stone; under identical conditions, the volume stability of modified aged steel slag (EAOR-ASS) was slightly superior to that of ASS, while the differences in high-temperature stability and low-temperature cracking resistance of the corresponding asphalt mixtures were minimal. Surface modification of ASS with EAOR significantly enhanced the water damage resistance of the asphalt mixture under freeze–thaw cycle conditions, thereby improving the utilization rate of steel slag. Both an increased volume replacement ratio of steel slag and surface modification with EAOR contributed to improved resistance to elastic deformation of the asphalt mixture.

## 1. Introduction

Road construction not only enhances regional connectivity but also plays a vital role in the economic development of countries worldwide [1]. Consequently, developing nations have invested substantial financial resources in the construction of transportation infrastructure over the past few decades [2]. However, excessive extraction of natural aggregates has led to a shortage of sand and gravel required for road construction and maintenance, resulting in rising costs and supply difficulties [3]. Steel slag, as one of the major solid wastes generated during steelmaking, is produced in China at an annual accumulation rate exceeding 100 million tons. Nevertheless, its comprehensive utilization rate remains significantly lower than that in developed European and American countries [4,5,6]. As a result, large quantities of steel slag have been stockpiled over the years, occupying vast areas of land and causing severe environmental pollution in surrounding regions [7,8]. On the other hand, the prolonged stockpiling of steel slag may result in groundwater contamination, which is attributed to the leaching of hazardous substances [9]. Therefore, promoting the engineering application of steel slag as a substitute for natural aggregates holds substantial economic value and environmental benefits [10,11,12].

Prior investigations have demonstrated that steel slag exhibits an irregular, angular morphology with a rough and highly porous surface, thereby endowing it with superior adhesion to bituminous binders [13,14]. Owing to its inherent hardness, steel slag has been widely recognized as a viable alternative to natural aggregates in asphalt mixtures [15,16]. Nevertheless, differential hydration kinetics of free lime (f-CaO) and free magnesia (f-MgO) in the presence of water provoke heterogeneous volumetric expansion and subsequent cracking of steel slag particles, thus compromising the in-service performance of asphalt mixtures incorporating such slag. A prevailing consensus in the literature attributes the volumetric instability of steel slag primarily to the presence of free lime [17,18,19]. To mitigate the deleterious influence of free lime (f-CaO) on the volumetric stability of steel slag, Huang et al. [20] subjected steel slag to oxalic-acid surface modification; the resulting slag exhibited enhanced physical characteristics and yielded asphalt mixtures whose fundamental pavement performance surpassed that of corresponding limestone-based mixes. Gao et al. [21] subsequently demonstrated that a 3% (mass fraction) oxalic-acid treatment significantly improved the volumetric stability of steel slag–asphalt mixtures relative to untreated controls, while simultaneously elucidating the underlying modification mechanisms. Zhang et al. [22], Liu et al. [23], and Sun et al. [24] further advanced the field by developing an integrated thermal-pressure-carbonation (TPC) process that exploits residual heat and a steam-laden environment to carbonate steel slag with CO_2_, thereby markedly reducing its expansion potential. Although both chemical modification and carbonation have proved highly effective in suppressing expansive reactions, the elevated technical demands and capital expenditures associated with carbonation facilities presently limit widespread deployment. Concurrently, natural weathering remains the predominant preconditioning strategy for steel slag stabilization worldwide [25,26]. In China, however, accelerating the utilization of historically stockpiled, weathered slags has become an urgent imperative driven by economic restructuring and stringent environmental regulations. Notwithstanding this imperative, systematic investigations into the combined effects of prolonged weathering and subsequent chemical surface modification on aggregate characteristics and the associated asphalt mixture performance remain conspicuously scarce.

Based on this, the present study employs cement paste (CP), silane coupling agent (SCA), polyvinyl alcohol solution (PVA), and epoxy acrylate-modified organosilicon resin (EAOR) to conduct surface modification on steel slag with particle size ranges of 4.75–9.5 mm and 9.5–13.2 mm. A comparative analysis of the aggregate characteristics of basalt and aged steel slag of the same particle size was conducted to determine the optimal surface modification process for aged steel slag. Furthermore, the road performance of three groups of asphalt mixtures, namely, basalt, aged steel slag, and modified aged steel slag, was evaluated under varying volumetric replacement ratios of steel slag. The findings aim to provide a theoretical and practical basis for the substitution of natural aggregates with aged steel slag and to support its large-scale engineering application.

## 2. Materials and Methods

### 2.1. Raw Materials

The steel slag used in this study was obtained from a steel plant located in Pingxiang City, Jiangxi Province, China. It had been stockpiled and aged for over one year, and its main chemical components are listed in Table 1. The steel slag samples had particle size ranges of 4.75–9.5 mm and 9.5–13.2 mm. The asphalt binder used was SBS-modified asphalt, designated as PG76-16, supplied by a company in Nanchang, Jiangxi Province, China and its performance indicators are presented in Table 2. Coarse and fine aggregates were selected as basalt and limestone chips, respectively, supplied by a company in Fuzhou, Jiangxi Province, China with their relevant performance indices provided in Table 3. The filler material used was limestone mineral powder, supplied by a company in Fuzhou, Jiangxi Province, China.

### 2.2. Experimental Scheme

**Figure 1:**
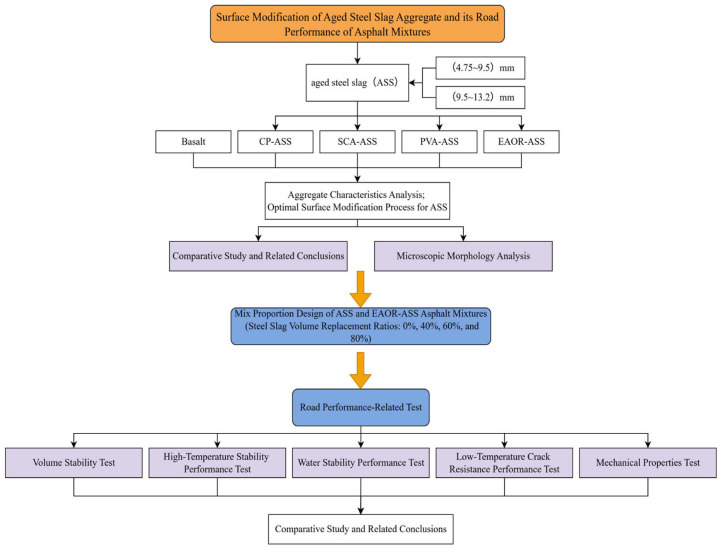
Overall Test Flowchart.

To investigate the characteristics of modified steel slag aggregates and their performance in asphalt mixtures, this study employed cement paste (CP), silane coupling agent (SCA), polyvinyl alcohol solution (PVA), and epoxy acrylate-modified organosilicon resin (EAOR) to conduct surface modification treatments on aged steel slag with particle size ranges of 4.75–9.5 mm and 9.5–13.2 mm. A comparative analysis of aggregate characteristics was performed at both macroscopic and microscopic levels between the modified steel slag and basalt of the same particle size, as well as unmodified aged steel slag, in order to determine the optimal surface modification process. Based on the above, the aged steel slag treated with the optimal surface modification process was used to replace basalt. Modified aged steel slag–asphalt mixtures with steel slag volume replacement ratios of 40%, 60%, and 80%, as well as unmodified aged steel slag–asphalt mixtures with the same replacement ratios, were compared with asphalt mixtures containing no aged steel slag. A comprehensive comparative analysis was conducted in terms of volume stability, high-temperature stability, moisture resistance, low-temperature crack resistance, and mechanical performance. The overall experimental process is shown in Figure 1. All performance tests shown in Figure 1 are required to meet the specified standard requirements [27]. The “standard requirements” referenced in subsequent tables and figures throughout this manuscript are derived from this standard.

### 2.3. Surface Modification Process for Aged Steel Slag

**Figure 2:**
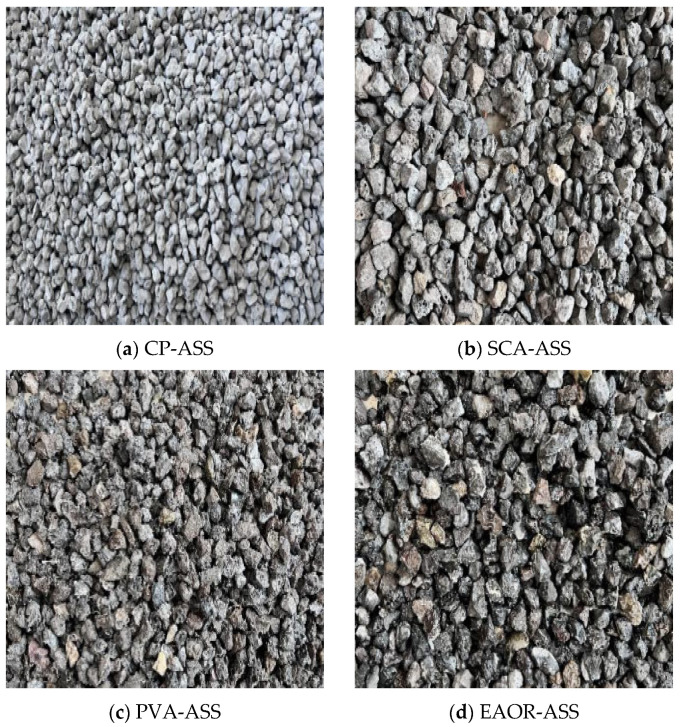
Four types of aged steel slag samples treated with surface modification by different substances.

To obtain modified aged steel slag (CP-ASS, SCA-ASS, PVA-ASS, and EAOR-ASS), surface modification treatments were applied to aged steel slag (ASS) with particle size ranges of 4.75–9.5 mm and 9.5–13.2 mm using cement paste (CP), silane coupling agent (SCA), polyvinyl alcohol solution (PVA), and epoxy acrylate-modified organosilicon resin (EAOR). The cement, supplied by a cement plant in Zhucheng, Shandong Province, China, was used to prepare cement paste at a water-to-cement ratio of 0.4. The surface modification of steel slag was conducted using an immersion method. The silane coupling agent KH-550, provided by a company in Nanjing, China, was hydrolyzed under optimal conditions with a mass ratio of m(KH-550):m(water):m(ethanol) = 5:45:50 and an optimal hydrolysis time of 20 min [28]. A KH-550 hydrolysate solution equivalent to 4% of the steel slag mass was then used to modify the steel slag surface via a blending method. PVA-1799 polyvinyl alcohol was provided by a company in Anhui Province, China. PVA solids were dissolved in boiling water to prepare an 8% (by mass) PVA solution, which was then used to treat the steel slag surface via an immersion method. EAOR was supplied by a company in Shandong Province, China. The EAOR modifying solution was prepared at a mass ratio of m(EAOR):m(xylene diluent) = 2:1 [29], and a quantity equivalent to 4% of the steel slag mass was blended with the slag to achieve surface modification. Figure 2 shows the four types of surface-modified aged steel slag samples. All photographs were captured using an ordinary smartphone camera under natural ambient lighting conditions.

### 2.4. Comparative Analysis of Aggregate Properties

#### 2.4.1. Macroscopic Physical Property Analysis

Macroscopic physical properties, including water absorption, crushing value, and adhesion grade between 70# asphalt and coarse aggregates, were investigated for basalt, ASS, CP-ASS, SCA-ASS, PVA-ASS, and EAOR-ASS with particle size ranges of 4.75–9.5 mm and 9.5–13.2 mm. The results are presented in Table 4.

As shown in Table 4, the water absorption of CP-ASS exceeds the specified limit, and the adhesion between PVA-ASS and 70# asphalt is relatively poor. The macroscopic physical properties of SCA-ASS are similar to those of ASS, but both are inferior to those of basalt. Only EAOR-ASS exhibits macroscopic physical properties comparable to those of basalt.

#### 2.4.2. Microscopic Morphology Analysis

1. Equipment Parameter Information

**Figure 3:**
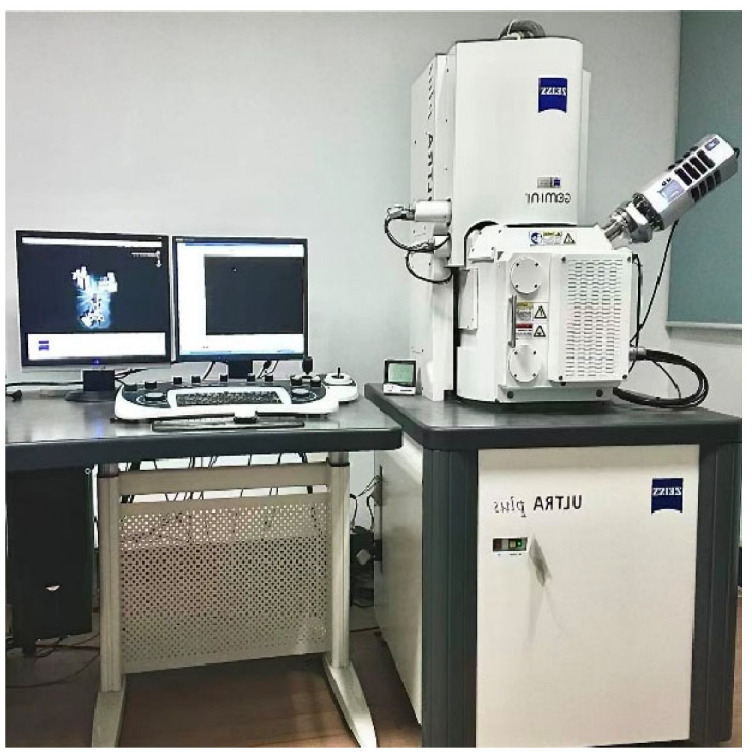
Zeiss Ultra Plus Scanning Electron Microscope (SEM).

Key parameters of the ZEISS Ultra Plus (ZEISS, Oberkochen, Germany) (Figure 3) field emission scanning electron microscope are listed below: Accelerating voltage: 0.1–30 kV; Beam current range: 4 pA–20 nA; Magnification: 12× to 400,000× (SE mode); Image resolution: 1.0 nm (15 kV, SE mode); EDS elemental detection range: Be4–U92; EDS resolution: Better than 127 eV (Mn Kα).

2. Sample Testing Procedures

(1) Wipe and clean the surface of the sample stage with alcohol cotton balls, then fix the cleaned sample stage on a foam board. Blow the sample stage with a clean air blower until it is completely dry, and subsequently attach conductive adhesive to the stage surface.

(2) Attach the steel slag specimens to the conductive adhesive on the sample stage. Cut a strip-shaped conductive adhesive to cover the specimen surface to ensure overall electrical conductivity of the samples and meet the conductive requirements for testing.

(3) Place the prepared samples into the scanning electron microscope for observation. Adjust parameters such as voltage, beam current, and contrast (detailed parameters are available in the SEM micrographs); focus on appropriate viewing areas; and capture images.

3. Analysis of the Microstructure of Samples

**Figure 4:**
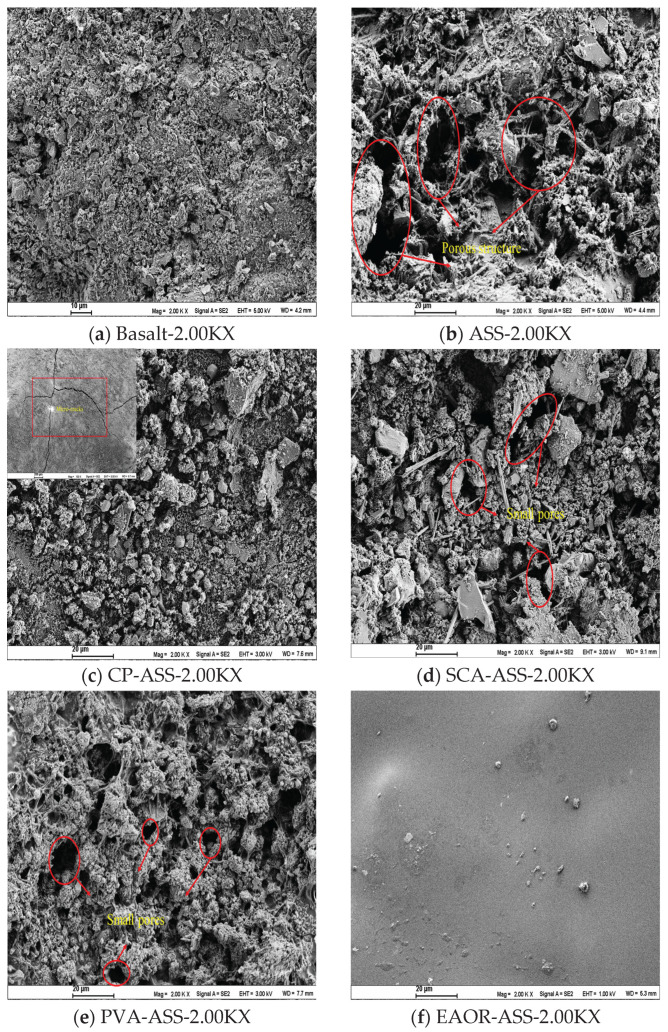
SEM-2.00KX-Microscopic Morphology Analysis.

As illustrated in Figure 4, under scanning electron microscopy (SEM) at a magnification of 2.00 KX, the surface of basalt exhibits relatively few pores and pronounced angularity. In contrast, ASS displays a markedly porous structure. Although the surface modification processes partially filled the pores in SCA-ASS and PVA-ASS, some voids remain visible. CP-ASS shows a relatively effective surface filling effect; however, due to the shrinkage characteristics of cement during drying, surface cracking occurs, resulting in higher water absorption. EAOR-ASS, on the other hand, is coated with a smooth film that not only effectively fills the surface voids of the steel slag but also preserves its angularity to a certain extent.

In summary, the surface modification of ASS with EAOR further enhances its aggregate properties. Compared with SCA-ASS, PVA-ASS, and CP-ASS, EAOR-ASS demonstrates superior performance and can therefore serve as a viable alternative to high-quality basalt aggregate.

### 2.5. Asphalt Mixture Design with Steel Slag

Using the gradation curve of the steel-slag-free asphalt mixture AC-13 as a reference, the aggregate blend gradation curves were designed for both EAOR-ASS–asphalt mixtures and ASS–asphalt mixtures with steel slag volume replacement ratios of 40%, 60%, and 80%. However, due to the significant differences in density among EAOR-ASS, ASS, basalt, and limestone chips, the conventional aggregate blend gradation curves—calculated by controlling the mass retained on standard sieves of various aperture sizes—deviate from the actual gradation of asphalt mixtures containing steel slag [30,31,32]. Therefore, the volumetric replacement method was employed to correct the gradation composition. The gradation composition and optimum asphalt–aggregate ratio for each group of steel-slag-incorporated asphalt mixtures were determined based on the results of Marshall tests. The relevant gradation designs are presented in Table 5, and the volumetric parameters are shown in Table 6.

### 2.6. Tests Related to the Road Performance of Asphalt Mixtures with Steel Slag

#### 2.6.1. Volume Stability Test

**Figure 5:**
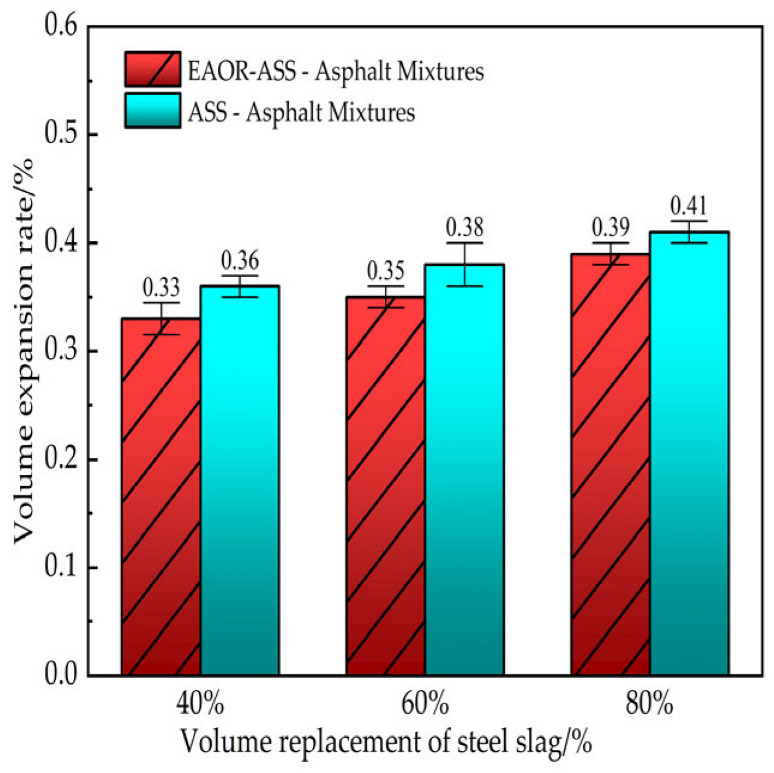
Volume Expansion Rates of Asphalt Mixtures with Steel Slag.

The volume expansion rate of steel-slag-incorporated asphalt mixtures under high-temperature water immersion is a critical indicator for evaluating their volume stability. A precision vernier caliper was employed to measure the diameters in three different orientations and the height of standard Marshall specimens prepared from both ASS–asphalt mixtures and EAOR-ASS–asphalt mixtures. The volume of the specimens at this stage was recorded as *V*_1_. After all specimens were measured, they were immersed in a constant-temperature water bath at 60 °C for 72 h. Subsequently, the same measurement method was applied to determine the post-immersion volume, denoted as *V*_2_. The volume expansion rate of the specimen, designated as *C*_1_, was calculated using Equation (1). The final result for each group was obtained by averaging the volume expansion rates from three parallel tests conducted under high-temperature water immersion conditions. The detailed experimental results are presented in Figure 5.

(1) $$C_{1}=\frac{V_{2}-V_{1}}{V_{1}}$$

#### 2.6.2. High-Temperature Stability Performance Test

**Figure 6:**
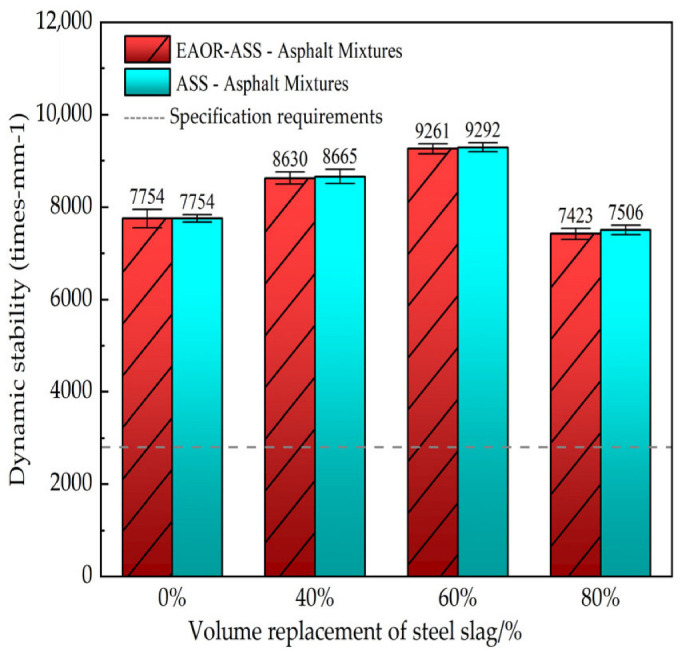
Rutting Test Results of Asphalt Mixtures with Steel Slag.

The resistance to deformation of asphalt mixtures without steel slag, as well as those incorporating ASS and EAOR-ASS at various volume replacement ratios, under the combined effects of high temperature and wheel loading was evaluated using the 60 °C wheel tracking test. This test was employed to assess the influence of steel slag replacement ratio and EAOR surface modification on the high-temperature stability of asphalt mixtures containing aged steel slag. According to the Standard Test Methods of Bitumen and Bituminous Mixtures for Highway Engineering (JTG E20-2011) [33], wheel tracking specimens with dimensions of 300 mm × 300 mm × 50 mm were tested in an automatic wheel tracking device at a temperature of 60 °C. A load of approximately 780 N was applied by the test wheel, which traversed the specimen at a frequency of 42 passes per minute for a duration of 1 h. The dynamic stability was calculated using Equation (2), and the test results are presented in Figure 6.

(2) $$DS=\frac{\left(t_{2}-t_{1}\right)}{d_{2}-d_{1}}\times N\times C_{1}\times C_{2}$$

In Equation (2), *DS* represents the dynamic stability of the asphalt mixture (cycles/min). A higher DS value indicates better high-temperature stability. The variables *d*_1_ and *d*_2_ denote the deformation values (in mm) of the specimen at time points *t*_1_ (45 min) and *t*_2_ (60 min), respectively. *C*_1_ and *C*_2_ are correlation coefficients, both taken as 1.0, and *N* refers to the reciprocating speed of the test wheel (cycles/min).

#### 2.6.3. Water Stability Performance Test

**Figure 7:**
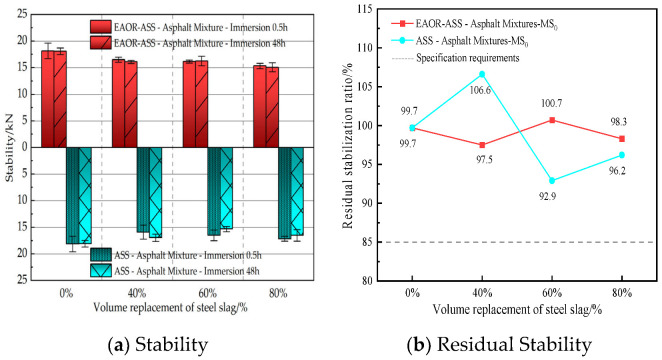
Results of Water-Soaked Marshall Stability Test.

**Figure 8:**
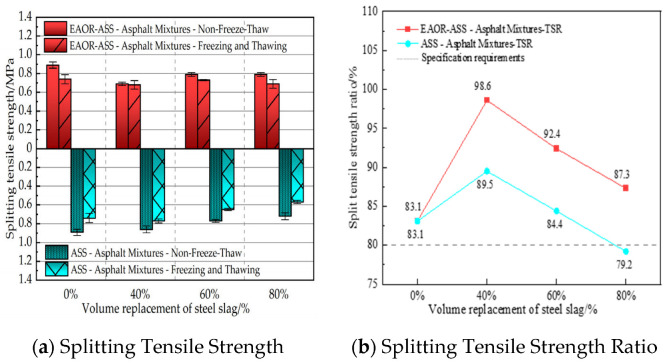
Results of Freeze–Thaw Splitting Test.

To evaluate the moisture resistance of asphalt mixtures without steel slag, as well as ASS–asphalt and EAOR-ASS–asphalt mixtures with different steel slag volume replacement ratios, under hot and humid summer conditions and winter freeze–thaw cycles, the water stability performance was investigated using the immersion Marshall stability test and the freeze–thaw splitting test. The residual Marshall stability of standard specimens (Φ101.6 mm ± 0.2 mm × 63.5 mm ± 1.3 mm) after immersion was calculated using Equation (3), and the tensile strength ratio after freeze–thaw cycling was calculated using Equation (4). The corresponding test results are presented in Figure 7 and Figure 8, respectively.

(3) $${MS}_{0}=\frac{{MS}_{1}}{MS}\times 100$$

In Equation (3), *MS*_0_ represents the residual Marshall stability after immersion, *MS*_1_ denotes the stability of standard Marshall specimens after being immersed in water at 60 °C for 48 h, and MS refers to the stability of specimens immersed for 30 min.

(4) $$TSR=\frac{\bar{R_{T2}}}{\bar{R_{T1}}}\times 100$$

In Equation (4), *TSR* represents the freeze–thaw splitting tensile strength ratio, where $\bar{{RT}_{2}}$ denotes the splitting tensile strength of standard Marshall specimens after freeze–thaw cycling, and $\bar{{RT}_{1}}$ denotes the splitting tensile strength of specimens not subjected to freeze–thaw cycling.

#### 2.6.4. Low-Temperature Crack Resistance Performance Test

**Figure 9:**
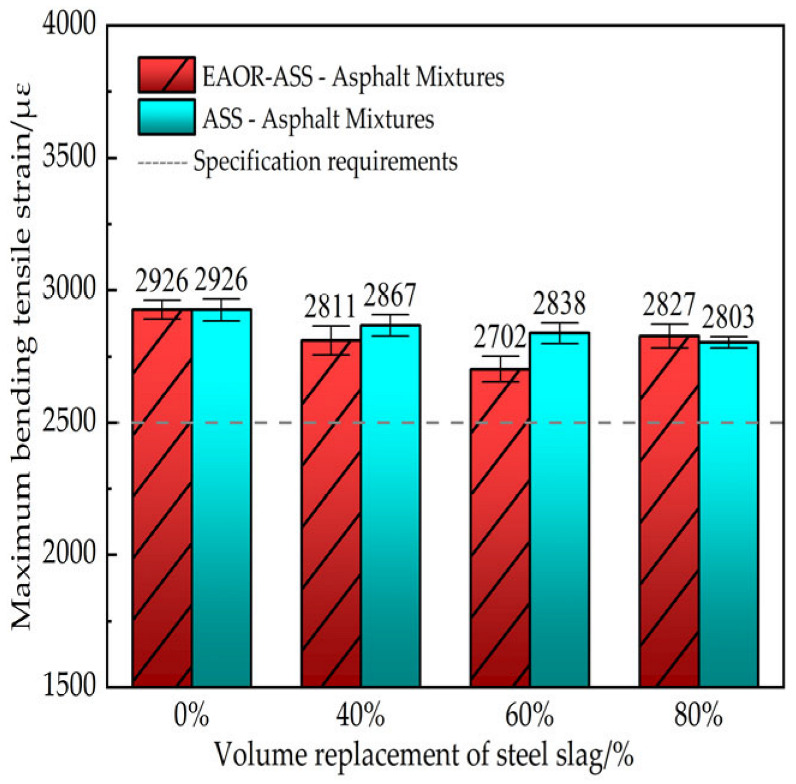
Maximum Bending Tensile Strain of Beam Specimens at −10 °C.

To investigate the low-temperature cracking resistance of asphalt mixtures without steel slag, as well as ASS–asphalt and EAOR-ASS–asphalt mixtures with different steel slag volume replacement ratios, beam specimens measuring 250 mm × 30 mm × 35 mm were subjected to bending tests at −10 °C. The maximum flexural tensile strain (με) at failure was calculated using Equation (5), and the test results are presented in Figure 9.

(5) $$\varepsilon_{B}=\frac{6\times h\times d}{L^{2}}$$

In Equation (5), $\varepsilon_{B}$ represents the maximum flexural tensile strain at failure of the beam specimen under a temperature of −10 °C, where *h* denotes the width of the cross-section at mid-span, and *d* is the mid-span deflection at the moment of failure.

#### 2.6.5. Mechanical Properties Testing

**Figure 10:**
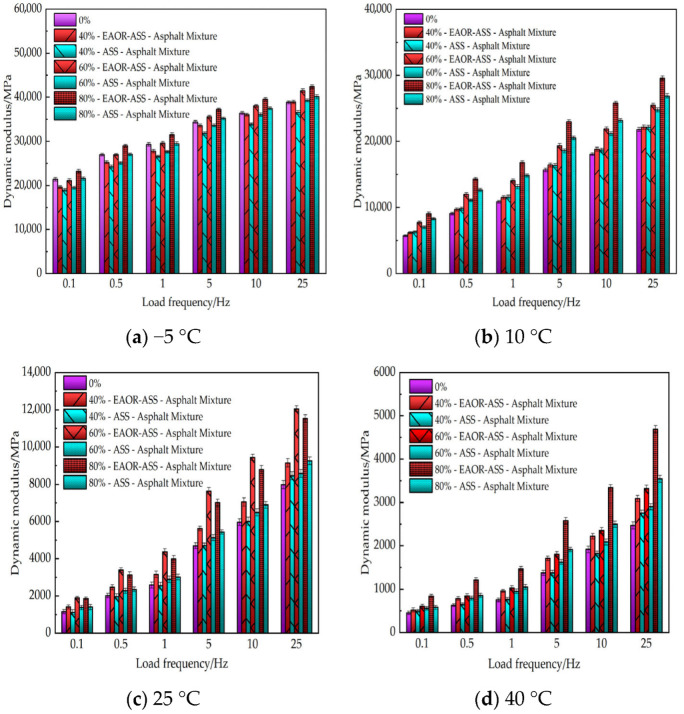
Results of Uniaxial Compression Dynamic Modulus Test.

As a typical viscoelastic material, asphalt mixture has a dynamic modulus that serves as a critical mechanical parameter in the structural design of asphalt pavements. Its magnitude is significantly influenced by environmental temperature and traffic loading conditions. Consequently, the elastic modulus obtained under a single temperature and loading frequency is insufficient to accurately reflect the actual mechanical state of asphalt pavements. To investigate the actual mechanical responses of asphalt mixtures without steel slag, as well as EAOR-ASS–asphalt and ASS–asphalt mixtures with different steel slag volume replacement ratios, specimens prepared using the Superpave Gyratory Compactor (SGC) were cored and trimmed. Dynamic modulus tests were then conducted using a UTM-25 testing system under multiple temperature conditions (−5 °C, 10 °C, 25 °C, and 40 °C) and various loading frequencies (0.1 Hz, 0.5 Hz, 1 Hz, 5 Hz, 10 Hz, and 25 Hz). The test results are presented in Figure 10.

**Figure 11:**
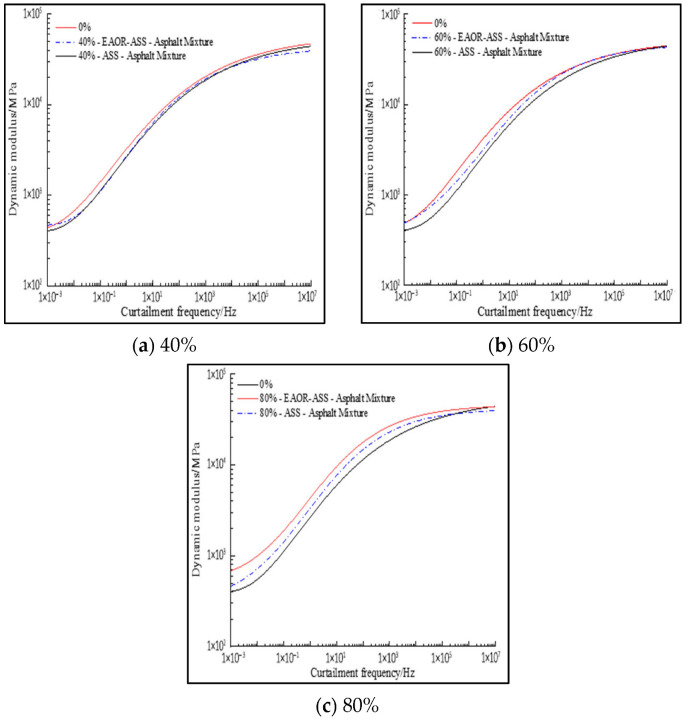
Comparison of Dynamic Modulus Master Curves.

To more accurately predict the actual mechanical performance of asphalt pavements under the combined effects of varying test temperatures, loading frequencies, steel slag volume replacement ratios, and surface modification treatments, 25 °C was selected as the reference temperature. Given that the dynamic modulus master curve generated by the standard sigmoidal function model is always symmetric about its inflection point—which may not be applicable to all types of asphalt mixtures—a generalized sigmoidal function model was adopted to construct the dynamic modulus master curve [34]. The generalized sigmoidal function is presented as Equation (6), the relationship between reduced frequency and loading frequency is given in Equation (7), and the temperature shift factor is calculated using Equation (8), where T and TR represent the test temperature and reference temperature, respectively. The relevant fitting parameters, including α, β, γ, δ, ε, *C*_1_, and *C*_2_, are listed in Table 7. To further investigate the differences in mechanical performance among EAOR-ASS–asphalt mixtures with varying steel slag volume replacement ratios, ASS–asphalt mixtures, and asphalt mixtures without steel slag, a comparative analysis of their dynamic modulus master curves was conducted, as illustrated in Figure 11.

(6) $$\mathrm{lg}\left(\left|E^{*}\right|\right)=\delta +\frac{\alpha }{{\left(1+\lambda e^{\beta +\gamma \cdot \mathrm{lg}f_{r}}\right)}^{\frac{1}{\lambda }}}$$

(7) $$\mathrm{lg}\left(f_{r}\right)=\mathrm{lg}f-\mathrm{lg}\left[a\left(T\right)\right]$$

(8) $$\mathrm{lg}\left[a\left(T\right)\right]=-\frac{C_{1}\left(T-T_{R}\right)}{C_{2}+\left(T-T_{R}\right)}$$

## 3. Results and Discussion

### 3.1. Volume Stability Test Results

As shown in Figure 5, the volume expansion rate (C_1_) increases with the increase in the volume replacement ratio of steel slag, which is attributed to the corresponding increase in the content of free calcium oxide (f-CaO) within the steel slag. Overall, the variation in C_1_ remains relatively small and well below the specified limit of 1.5%, indicating that the volume stability of the mixtures is satisfactory. This is primarily due to the gradual reduction of f-CaO content in aged steel slag (ASS) under prolonged exposure to natural weathering conditions such as sunlight and rainfall. Furthermore, the volume expansion rate of ASS–asphalt mixtures is slightly higher than that of EAOR-ASS–asphalt mixtures, suggesting that surface modification of ASS with EAOR can effectively enhance the volume stability of the resulting asphalt mixtures.

### 3.2. High-Temperature Stability Performance Test Results

As shown in Figure 6, the dynamic stability values initially increase and then decrease with the rising volume replacement ratio of steel slag. This trend can be attributed to the superior strength and angularity of aged steel slag (ASS) compared to basalt, which enhances the high-temperature stability of asphalt mixtures when ASS partially replaces basalt. However, when the replacement ratio becomes excessively high, the sharp edges and corners of steel slag particles may reduce the interlocking effect between aggregates. Consequently, the strength of the asphalt mixture becomes more dependent on the adhesion between the asphalt mastic and the aggregate particles. Since asphalt is a viscoelastic material, the asphalt mixture with an 80% volume replacement ratio of aged steel slag exhibits reduced resistance to deformation under the combined effects of high temperature and wheel loading. Under the same volume replacement ratio, the optimum asphalt–aggregate ratio of ASS–asphalt mixtures differs from that of EAOR-ASS–asphalt mixtures by approximately 0.4%. However, the difference in their dynamic stability values is relatively minor. This can be attributed to the fact that the surface of EAOR-ASS is thoroughly filled by EAOR, which is equivalent to the complete coating of ASS by asphalt. Consequently, the difference in adhesion between EAOR-ASS or ASS and the asphalt binder is minimal.

### 3.3. Water Stability Performance Test Results

As shown in Figure 7, the residual stability (*MS*_0_) values do not exhibit a clear trend of variation with increasing steel slag volume replacement ratio. However, all asphalt mixture groups demonstrate favorable resistance to moisture damage. When the steel slag volume replacement ratio reaches 60% and 80%, the EAOR-ASS–asphalt mixtures exhibit superior resistance to moisture damage under prolonged high-temperature water immersion compared to ASS–asphalt mixtures. This indicates that surface modification of ASS with EAOR contributes to enhancing the moisture stability of asphalt mixtures containing steel slag.

As shown in Figure 8, asphalt mixtures with a steel slag volume replacement ratio of 40% exhibit higher TSR values compared to those without steel slag. This is attributed to the rougher surface texture of steel slag compared to basalt, which enhances its adhesion to asphalt. However, when the steel slag volume replacement ratio exceeds 40%, the splitting tensile strength of ASS–asphalt mixtures decreases significantly with increasing replacement ratio. This is due to the higher water absorption of steel slag compared to basalt; as the volume replacement ratio increases, the overall water absorption of the ASS–asphalt mixture rises accordingly. Consequently, components such as free calcium oxide (f-CaO) have sufficient conditions to react with retained moisture, leading to expansion reactions. These reactions induce internal stress accumulation within the asphalt mixture, and freeze–thaw cycling further exacerbates stress concentration, resulting in structural damage to the ASS–asphalt mixture. Under the same conditions, the TSR values of EAOR-ASS–asphalt mixtures are significantly higher than those of ASS–asphalt mixtures, indicating that surface modification of ASS with EAOR effectively enhances the moisture resistance of steel-slag-incorporated asphalt mixtures under harsh environmental conditions.

### 3.4. Low-Temperature Crack Resistance Performance Test Results

As shown in Figure 9, the maximum flexural tensile strain of asphalt mixtures containing steel slag is slightly lower than that of mixtures without steel slag. This is attributed to the presence of small surface pores on the beam specimens after cutting, which somewhat reduces their flexural tensile capacity. Overall, there is little difference in low-temperature crack resistance between EAOR-ASS–asphalt mixtures and ASS–asphalt mixtures, indicating that surface modification of ASS with EAOR does not significantly enhance the low-temperature cracking performance of the resulting asphalt mixtures.

### 3.5. Mechanical Properties Testing—Uniaxial Compression Dynamic Modulus Test Results

Figure 10 presents the results of the uniaxial compressive dynamic modulus test. The test objects were EAOR-ASS–asphalt mixtures with different volume replacement ratios and ASS–asphalt mixtures. The test primarily considered different test temperatures and loading frequencies, which provides a certain reference value for demonstrating the mechanical properties of actual asphalt pavements. However, there are still some differences compared to the actual traffic load coupling conditions in real asphalt pavements.

As shown in Figure 10, the dynamic modulus increases with the increase in the volume replacement ratio of steel slag, and the values for EAOR-ASS–asphalt mixtures are higher than those for ASS–asphalt mixtures. This is attributed to the superior angularity and higher strength of steel slag compared to basalt. Additionally, surface modification of ASS with EAOR not only effectively fills the aged steel slag surface pores but also enhances the adhesion between asphalt and steel slag, resulting in stronger bonding between asphalt and EAOR-ASS. As the test temperature increases and the loading frequency becomes higher, the dynamic modulus of EAOR-ASS–asphalt mixtures is greater than that of both ASS–asphalt mixtures and asphalt mixtures without steel slag. This indicates that surface modification of ASS with EAOR can enhance the resistance to elastic deformation of the resulting asphalt mixtures under high-temperature and high-loading-frequency conditions.

As illustrated in Figure 11, the dynamic modulus increases with the increase in reduced frequency. This phenomenon can be attributed to the fact that an increase in reduced frequency is equivalent to a decrease in environmental temperature, thereby enhancing the overall elastic response of the test specimens. Under conditions of extremely high reduced frequency, the dynamic moduli of the EAOR-ASS–asphalt mixture, the ASS–asphalt mixture, and the asphalt mixture without steel slag are nearly identical. This indicates that, under extreme environmental conditions, the dynamic modulus of asphalt mixtures is independent of both the surface modification process of steel slag and the volumetric replacement ratio of steel slag.

## 4. Conclusions

In order to promote the utilization of aged steel slag as a substitute for high-quality natural aggregates and to provide a reference for its engineering applications, the authors conducted surface modification treatments on aged steel slag. Subsequently, laboratory investigations were carried out on the road performance of EAOR-ASS–asphalt mixtures, ASS–asphalt mixtures, and asphalt mixtures without steel slag, each prepared with different volumetric replacement ratios of steel slag. The following conclusions were drawn:

1. Surface modification of ASS using EAOR can yield EAOR-ASS with properties comparable to those of basalt aggregates.

2. The volume expansion rate of steel slag–asphalt mixtures increased with the steel slag replacement ratio. At 80% replacement, the EAOR-ASS and ASS mixtures exhibited volume expansion rates of 0.39% and 0.41%, respectively, both well below the 1.5% specification limit. The EAOR surface modification slightly reduced the expansion rate compared to unmodified ASS at equivalent replacement ratios.

3. The rutting resistance of EAOR-ASS–asphalt mixtures is comparable to that of ASS–asphalt mixtures. When the steel slag volume replacement ratio is ≤60%, the dynamic stability values of the asphalt mixtures are significantly higher than those of mixtures without steel slag. However, an excessively high steel slag replacement ratio may weaken the interlocking force between aggregates in the asphalt mixture, resulting in a decrease in dynamic stability.

4. Compared with both the asphalt mixture without steel slag and the ASS–asphalt mixture, the EAOR-ASS–asphalt mixture exhibits superior resistance to moisture damage. Additionally, it allows for a reduction in asphalt binder content and an increase in the volume replacement ratio of steel slag.

5. Overall, the maximum flexural tensile strength of asphalt mixtures incorporating steel slag is slightly lower than that of mixtures without steel slag. Moreover, there is a relatively small difference in low-temperature crack resistance between EAOR-ASS–asphalt mixtures and ASS–asphalt mixtures.

6. The incorporation of steel slag can enhance the resistance to elastic deformation of asphalt mixtures, and surface modification of ASS using EAOR can further improve this performance. However, under ultra-high reduced frequency conditions, the dynamic modulus of asphalt mixtures is independent of both the surface modification process of aged steel slag and the volume replacement ratio of steel slag.

## Figures and Tables

**Table 1:** Main Components of Aged Steel Slag.

Chemical Composition	SiO_2_	Al_2_O_3_	CaO	MgO	Fe_2_O_3_	P_2_O_5_	MnO	Other
Content	19.4%	5.16%	34.1%	9.47%	16.5%	2.36%	7.77%	5.24%

**Table 2:** Main Performance Indicators of SBS-Modified Asphalt.

Test Item		Specification Requirements	Test Results
Penetration (25 °C, 5 s, 100 g)/0.1 mm		40~60	55.0
Ductility (5 °C, 5 cm/min)/cm		≥20	32.0
Softening Point/℃		≥60	81.0
Flash Point/℃		≥230	295
Elastic Recovery (25 °C)/%		≥75	91.8
RTFOT	Mass Change/%	≤±1.0	−0.164
	Penetration Ratio/%	≥65	74.8
	Ductility (5 °C)/cm	≥15	16

**Table 3:** Main Performance Indicators of Coarse and Fine Aggregates.

Test Item	Specification Requirements	Basalt		Limestone Chips	
		10~15 mm	5~10 mm	3~5 mm	0~3 mm
Crushing Value/%	≤26	21.50		—	—
Content of Flaky and Elongated Particles/%	≤12	—	9.1	—	—
Los Angeles Abrasion Loss/%	≤30	20.20		—	—
Water Absorption/%	≤2.0	0.41	0.57	0.65	0.88
Sand Equivalent/%	≥65	—	—	—	68
Angularity/%	≥30	—	—	—	39

**Table 4:** Macroscopic Physical Properties of Basalt and Steel Slag.

Aggregate	Water Absorption/%	Crushing Value/%	Adhesion Grade with Asphalt	Apparent Relative Density	Bulk Relative Density
Basalt	0.41	21.50	5	2.861	2.828
ASS	1.28	23.81	5	3.308	3.173
CP-ASS	4.11	23.31	5	3.184	2.816
SCA-ASS	1.17	22.57	5	3.302	3.180
PVA-ASS	1.13	17.55	3	3.224	3.111
EAOR-ASS	0.47	18.97	5	3.174	3.128
Specification Requirements	≤2.0	≤26	≥4	—	—

**Table 5:** Gradation Design of Asphalt Mixtures with Steel Slag.

Mixture Type	Steel Slag Type	Steel Slag Volume Replacement of Basalt	Optimal Asphalt-to-Aggregate Ratio	Basalt		Limestone Chips		Mineral Powder	Aged Steel Slag	
				10~15 mm	5~10 mm	3~5 mm	0~3 mm		9.5~13.2 mm	4.75~9.5 mm
I	—	0%	4.9	28	27	11	32	2	0	0
II	EAOR-ASS	40%	4.7	16	15.3	11	32	2	12	11.7
III	ASS	40%	5.1	16	15.3	11	32	2	12	11.7
IV	EAOR-ASS	60%	4.8	10.4	10	11	32	2	17.6	17
V	ASS	60%	5.2	10.4	10	11	32	2	17.6	17
VI	EAOR-ASS	80%	4.8	5.1	4.9	11	32	2	22.9	22.1
VII	ASS	80%	5.2	5.1	4.9	11	32	2	22.9	22.1

**Table 6:** Volumetric Parameters of Asphalt Mixtures with Steel Slag.

Mixture Type	Bulk Relative Density	Void Ratio/%	Stability/kN	Flow Value/0.1 mm	Aggregate Void Ratio/%	Asphalt Saturation/%
I	2.461	4.1	18.15	3.45	14.2	71.4
II	2.518	4.2	16.10	3.31	14.2	70.1
III	2.510	4.4	16.96	3.26	14.9	70.5
IV	2.545	4.1	16.28	3.33	14.3	71.3
V	2.551	4.2	15.33	3.10	14.9	71.9
VI	2.570	4.1	15.09	3.08	14.4	71.5
VII	2.575	4.1	16.52	3.27	14.9	72.3

**Table 7:** Fitting Parameters for the Generalized Sigmoidal Function.

Mixtur Type	Steel Slag Type	Steel Slag Volume Replacement of Basalt	δ	α	β	γ	λ	C_1_	C_2_	R^2^
I	—	0%	2.60	2.14	0.17	0.42	0.26	13.58	123.15	0.99
II	EAOR-ASS	40%	2.61	2.16	0.23	0.41	0.22	17.93	167.71	0.99
III	ASS	40%	2.67	1.98	0.14	0.49	0.20	15.50	143.27	0.99
IV	EAOR-ASS	60%	2.63	2.09	0.37	0.43	0.22	22.88	193.56	0.99
V	ASS	60%	2.50	2.17	0.01	0.58	0.60	21.48	191.85	0.99
VI	EAOR-ASS	80%	2.71	1.95	0.08	0.65	0.55	13.63	133.44	0.98
VII	ASS	80%	2.47	2.15	0.06	0.65	0.74	12.63	118.18	0.98

## Data Availability

The data presented in this study are available on request from the corresponding author due to privacy protection restrictions.

## Abbreviations

The following abbreviations are used in this manuscript:

EAOR	Epoxy Acrylate-Modified Organosilicon Resin
EAOR-ASS	Epoxy Acrylic-Modified Silicone Resin-Modified Aged Steel Slag
ASS	Aged Steel Slag
CP	Cement Paste
SCA	Silane Coupling Agent
PVA	Polyvinyl Alcohol Solution
